# Science: our past, our present, and always our future

**DOI:** 10.1172/jci.insight.193355

**Published:** 2025-04-08

**Authors:** Oliver Eickelberg, Christopher S. Williams, Kyu Y. Rhee

Physician-scientists serve as the backbone, constituency, and inspiration for the American Society for Clinical Investigation (ASCI) and the ASCI Family of Journals, the *Journal of Clinical Investigation* (*JCI*) and *JCI Insight*. The Editorial Board of *JCI Insight* is honored to continue and expand upon this tradition by dedicating a collection of Physician-Scientist Development articles that feature editorials, research communications, and reviews on the life, work, challenges, and aspirations of physician-scientists. This collection includes an exciting review on the history of the physician-scientist by former ASCI president Gary Koretzky (1), a perspective on the history and goals of the ASCI/Alliance for Academic Internal Medicine/Burroughs Wellcome Fund (ASCI/AAIM/BWF) Physician-Scientist Pathways Workshop (2), an evaluation of the effect of pre-health advising on the MD-PhD applicant pool (3), and an investigation into whether application metrics are able to predict physician-scientist outcomes of success (4).

The beginnings of the ASCI are commonly attributed to the activity and ambition of Samuel James Meltzer (1851–1920), a physician-scientist born in Curland (northwestern Russia) and educated in Berlin (Germany), who ultimately rose through the academic ranks at the Rockefeller Institute for Medical Research in New York (later to become Rockefeller University). Dr. Meltzer was among the first to study esophageal manometry and is remembered as a physician-scientist who was eager to transform medical research from a static, autopsy-based clinicopathological view to a dynamic, physiology-based disease view using the latest laboratory techniques. This ambition drove the founding of the ASCI in 1909.

Dr. Meltzer not only volunteered for his own studies, usually by placing two stomach tubes in his esophagus to study swallowing dynamics, but also was a highly respected and sought-after mentor. He demonstrated that the “relationship between student and professor was not that of a poor dependent and well-to-do patron, but an intellectual companionship of two scholarly men not influenced by the mere externalities of life” (5).

To this day, mentorship relations remain at the core of every successful physician-scientist’s career. Although we have moved on from the single-mentor/mentee paradigm to more team-based mentor/sponsor/mentee relationships and peer mentoring groups, successfully navigating the academic career landscape without such support teams remains a daunting journey. Every one of us can easily name multiple occasions where a peer, sponsor, or mentor had a significant impact on our career, very positive most times, but sometimes less so.

Many of us remember the very first time when, as a young, enthusiastic, and hopeful trainee, we were introduced to a leading scientist in our field, at a conference, at an invited seminar, or over coffee. Many of us have stories to tell about how grateful we are for that small, seemingly irrelevant piece of key advice that ended up being highly relevant to our career. Yet many of us also have stories about how our view of our mentor(s) changed over time, especially as scientific approaches carved out from a mentor’s research program needed to fuel one’s own career. Whether we remember those moments as incredibly positive or eternally painful, collectively they allowed us to grow as people and scientists, highlighting the invaluable roles mentors, peers, and sponsors play in our lives.

In 1925, Harry Sinclair Lewis published the Pulitzer Prize–winning tale of Martin Arrowsmith of the fictional town of Elk Mills in the fictional state of Winnemac (6). Arrowsmith is the archetype of the physician-scientist, who struggled to navigate competing philosophical and practical tensions of clinical medicine and scientific research. Lewis went on to become the first US citizen to receive the Nobel Prize in Literature “for his vigorous and graphic art of description and his ability to create, with wit and humour, new types of characters” (7). The somewhat startling timelessness of Lewis’s portrayal of the physician-scientist career is due, in part, to an overly romanticized view of physician-scientists by academic medical centers, as individuals engage in full-time careers in both clinical practice and scientific discovery. The challenges of modern health care delivery and biomedical research, however, have evolved into complex enterprises that have increasingly outmatched the time and resources needed to pursue both career facets simultaneously.

In light of this growing dissociation, it is both instructive and timely to review the actual history of physician-scientists and their landmark contributions over past decades/centuries. Such a view is not merely a semantic shift — it is a radical reimagining of the physician-scientist career as both a profession and a vocation. For academic medical centers, this new paradigm opens intriguing possibilities. Rather than physician-scientists generating both clinical relative value units and conventional research metrics, funding models that embrace the unique bridging role of the physician-scientist should be developed. Some forward-thinking centers are experimenting with hybrid support structures that combine clinical revenue, research funding, and institutional investment in translation. These models recognize that the value of physician-scientists extends far beyond traditional metrics — they serve as institutional innovation engines, attracting research funding through their distinctive perspective on translational opportunities and enhancing institutional prestige through high-impact discoveries.

As we mark the centennial of Lewis’s *Arrowsmith*, it is time for physician-scientists to take stock and visibly and vocally advocate for what we do best: generate and test key hypotheses that will alter the face of clinical medicine. This will enable the physician-scientist career path to be redeveloped in a systematically optimized and pedagogically, operationally, and financially integrated manner. We believe that through the sustained efforts of organizations such as the AAIM, ASCI, and BWF, alongside forward-thinking academic medical centers and dedicated forums, including *JCI Insight*, we can establish career paths that are both achievable and sustainable. Success will require more than just redefining roles — it will demand reimagining how our institutions support and harness these unique individuals, who serve as crucial bridges between scientific discovery and clinical care. Ultimately, we will need a concerted and strategic effort to communicate all of the above to the public and help the public gain trust in innovative, significant, and rigorous scientific discovery, again. The future of medical innovation may well depend on it.

Importantly, our goal at *JCI Insight* is to provide evidence- and history-based data and facts but also inspiration from generations of physician-scientists to share with our early-career individuals. We would like to remind those of us at later career stages about the crucial importance of positive mentorship. Our path has never been easy or well laid out and is paved with failure, hardship, long nights, failed experiments, and at times, suffering of personal relationships. It has always been and will always be the less traditional, more cumbersome, but ultimately most exciting and fulfilling career trajectory.

Importantly, the course of the physician-scientist will not become easier in years ahead. Uncertainty about financial viability of this career path will undoubtedly lead to many future trailblazers abandoning their aspirations. The important message in these times is that you are not alone. You have a home, and this is the ASCI and her journals. We will do our utmost to ensure the long-term career sustainability of physician-scientists. Go, flight!

## Version 1 04/08/2025

Electronic publication

## References

[1] Koretzky G (2025). The physician-scientist: looking back, looking forward. JCI Insight.

[2] Rhee KY (2025). Paving the physician-scientist career path: from grassroots gathering to national forum. JCI Insight.

[3] Plaza-Jennings AL (2025). A study of MD-PhD pre-health advising identifies challenges to building a robust MD-PhD applicant pool. JCI Insight.

[4] Brass LF (2025). Admissions to MD-PhD programs: how well do application metrics predict short- or long-term physician-scientist outcomes?. JCI Insight.

[7] https://www.nobelprize.org/prizes/literature/1930/lewis/facts/.

